# Central diabetes insipidus: a late sequela of BNT162b2 SARS-CoV-2 mRNA vaccine?

**DOI:** 10.1186/s12902-023-01296-4

**Published:** 2023-02-22

**Authors:** Avraham Ishay, Elena Chertok Shacham

**Affiliations:** 1 Endocrinology Unit, HaEmek Medical Center, Yitzhak Rabin Av. 21, 18101 Afula, Israel; 2 Faculty of Medicine, Technion – Israel Institute of Technology, Haifa, Israel

**Keywords:** COVID-19 vaccine, Diabetes Insipidus, Hypophysitis, SARS-CoV-2, Case report

## Abstract

**Background:**

The development of an effective vaccine is a powerful tool to contain the global spread of coronavirus disease 2019 (COVID-19). Still, it raises potential safety concerns about the subsequent enhancement of associated immunopathology. Increasing evidence shows that the endocrine system, including the hypophysis, may be involved in COVID-19. Moreover, occasional but increasing reports of endocrine disorders involving the thyroid have been reported after the severe acute respiratory syndrome coronavirus 2 (SARS-CoV-2) vaccine. Among them, a few cases encompass the pituitary. Here we report a rare case of central diabetes insipidus following SARS-CoV-2 vaccination.

**Case presentation:**

We report a 59-year-old female patient with a 25-year history of Crohn's disease in long-term remission, who presented with sudden onset of polyuria eight weeks after administration of an mRNA SARS-CoV-2 vaccination. Laboratory evaluation was consistent with isolated central diabetes insipidus. Magnetic resonance imaging displayed involvement of the infundibulum and the posterior hypophysis. Eighteen months after the vaccination, she is still under desmopressin treatment and had stable pituitary stalk thickening on magnetic resonance imaging. Although Crohn's disease-associated hypophysitis has been reported, it is scarce. In the absence of other recognizable causes of hypophysitis, we believe the involvement of the hypophysis in our patient may have been triggered by the SARS-CoV-2 vaccine.

**Conclusions:**

We report a rare case of central diabetes insipidus potentially associated with SARS-CoV-2 mRNA vaccination. Further studies are needed to understand better the mechanisms underlying autoimmune endocrinopathies development in the context of COVID-19 infection and SARS-CoV-2 vaccination.

## Background

As is well known, severe acute respiratory syndrome coronavirus 2 (SARS-CoV-2) has affected tens of millions of people and resulted in more than 3 million deaths worldwide [1]. The emergence of this pandemic led to the use of novel technologies and the development of SARS-CoV-2 vaccines. The different types of COVID-19 vaccines are mRNA-based (BNT162b2/ Pfizer, mRNA-1273/Moderna), viral vector vaccines (Ad26.CoV2.S; Johnson & Johnson, ChAdOx; AstraZeneca), inactivated (Sinopharm, Sinovac) and protein subunit vaccines (Novavax) [2]. Full vaccination dose, younger age, female sex, and vaccine brand (e.g., mRNA-1273 more frequently than BNT162b2) were shown to be associated with an increased risk of adverse events [3]. Endocrine adverse effects were reported following all types of vaccines, but pituitary adverse events were reported only after mRNA and adenovirus vector vaccines [4]. The SARS-CoV-2 virus enters the host cells through the angiotensin-converting enzyme 2 (ACE2) receptor and the transmembrane serine protease 2 (TMPRSS2) protein widely expressed in endocrine glands. This led to the vulnerability of the endocrine system during the acute and post-acute phases following SARS-CoV-2 infection [5, 6]. The most reported endocrine perturbations from COVID-19 are thyroid dysfunction, and hyperglycemia [6] but early and late pituitary harm after COVID-19 illness, including central diabetes insipidus and pituitary apoplexy have been reported [7–9]. Inversely, hyponatremia in COVID-19 patients has been related to possible inappropriate antidiuretic hormone secretion [5] Hypophysitis is a generic term that includes heterogeneous conditions that cause inflammatory infiltration of the pituitary gland, potentially resulting in hormonal deficiencies and mass effects. It can be classified according to the anatomic location of the pituitary involvement and the cause: idiopathic or secondary to neoplastic lesions of the sellar/suprasellar region, autoimmune, inflammatory, or proliferative disorders and drugs (mainly immune checkpoint inhibitors in the last years). Lymphocytic hypophysitis is the most common histologic variant of primary hypophysitis [10]. Recently, two cases of central diabetes insipidus have been reported after SARS-CoV-2 vaccination [4, 11].

Herein, we report a novel case of infundibulo-neurohypophysitis presenting with isolated diabetes insipidus as a possible late-onset sequela of the mRNA-based BNT162b2 vaccination.

## Case presentation

A 59-year-old nurse presented in the endocrinology unit of our hospital following two weeks of suffering from polyuria, increased thirst, fatigue, and a 6-pound weight loss. A 24-h urine volume was 10 L. She had a long-standing history of Crohn's disease, well controlled with mesalamine. Eight weeks before the onset of symptoms, she received the first dose of the Pfizer—BioNTech COVID -19 vaccine. Erythema and mild pain at the injection site resolved within 72 h. Eight weeks post-vaccination, anti-spike SARS-CoV-2 IgG titers were 3004 UA/ml (cut-off 50 UA/ml) using chemiluminescent immunoassay, Abbott Architect.

Her physical examination was unremarkable except for mild dehydration, dry skin, and pallor. There were no visual disturbances. After overnight dehydration, her serum sodium was 148 mEq/L, and her blood osmolarity was 318 mOsm/Kg. Urine osmolarity and sodium were low- 100 mOsm/Kg and 10 mEq/L, respectively. One hour after IV administration of 2 µg desmopressin, the patient showed clinical improvement: a decrease in urine output, an increase in urine osmolarity from100 mOsm/kg to 530 mOsm/kg, and her serum sodium and serum osmolarity returned to normal.

**Fig. 1:**
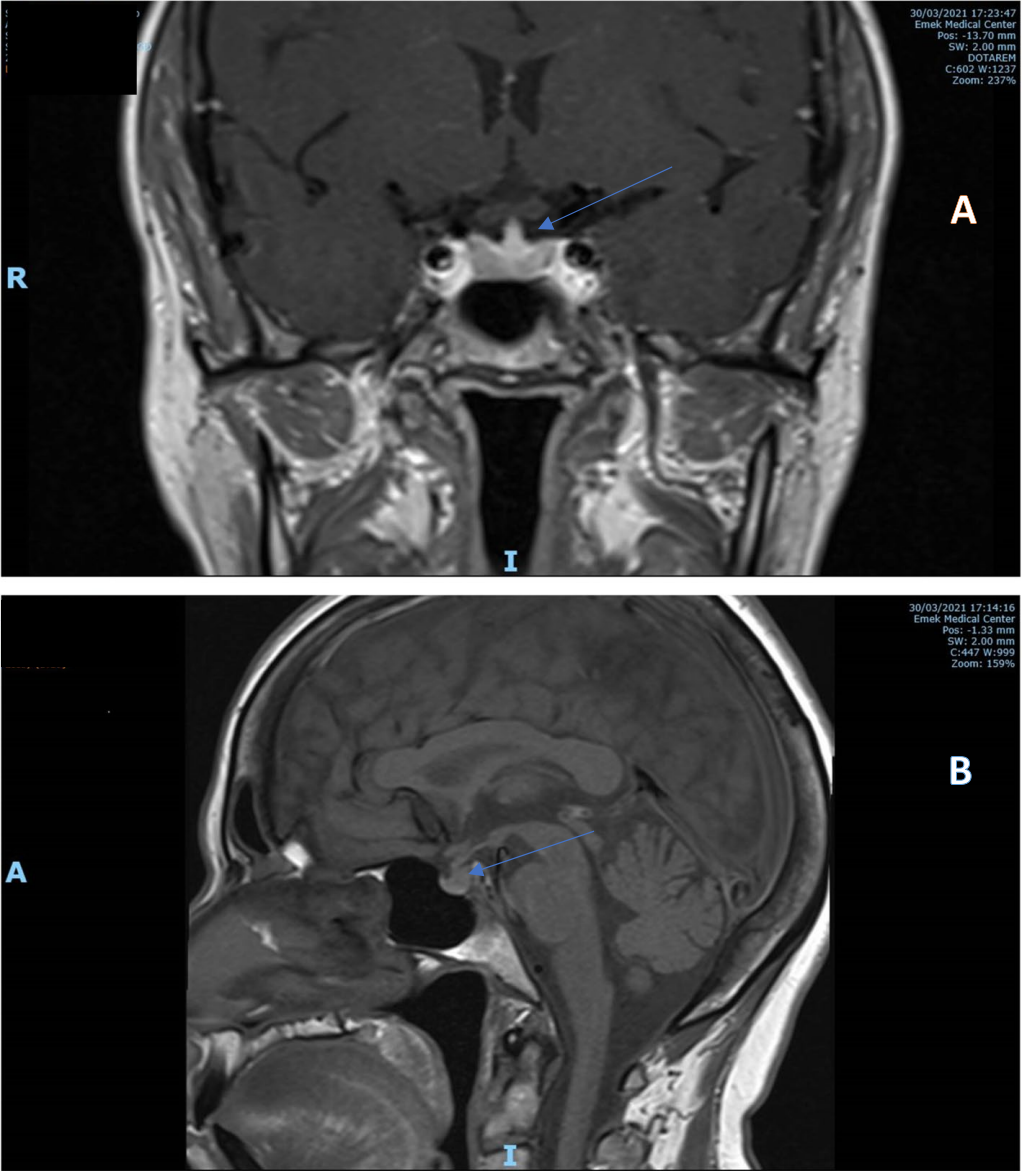
T1 weighted magnetic resonance (MR) images. **A** Coronal view after contrast medium injection showing thickening of the pituitary stalk (arrow). **B** Sagittal view before contrast medium injection shows the loss of the bright spot of neurohypophysis (arrow)

Magnetic resonance imaging of the sella region displayed a thickened pituitary stalk without sellar or suprasellar masses (Fig. 1A). In addition, the bright spot of the neurohypophysis was not visible (Fig. 1B).

Laboratory evaluation showed a Glomerular filtration rate (GFR) of 76 ml/min, calcium 9.6 mg/dl, glucose 87 mg/dl, and Angiotensin-converting enzyme 54 U/l (19,8–70.2 U/l). The human chorionic gonadotropic level was undetectable. Assessment of the anterior pituitary function revealed normal levels of thyroid stimulating hormone (TSH) 2.2 µIU/ml (0.4–4.2), Free thyroxine (FT4) 14.7 pmol/l (11.5–22.7), Adrenocorticotropic hormone (ACTH) 20.5 pg/ml (0–46), morning cortisol level of 16.9 µg/dl, prolactin 9.2 ng/ml (2.8–29 ng/ml), and an Insulin-like Growth Factor 1 (IGF1) level of 155 ng/ml (43–220), along with gonadotropins levels in the post-menopausal range. Her IgG4 level was 15 mg/dl (3.9–86 mg/dl). The antidiuretic hormone level was undetectable: < 2 pg/mL (0.5–8). Anti-nuclear antibodies (ANA) and extractable nuclear antigen (ENA) antibodies were negative. Serum complement levels C3 and C4 were normal.

The presentation with isolated diabetes insipidus without structural or hormonal involvement of the anterior hypophysis was consistent with the diagnosis of infundibuloneurohypophysitis. Treatment with oral desmopressin 0.2 mg twice daily was started with a rapid resolution of symptoms. After three days, the patient’s urine volume was reduced to 1.5 L/d, and her electrolytes and osmolarity in blood and urine were within normal limits. Eighteen months after vaccination the patient is still under desmopressin therapy.

## Discussion and conclusions

Central diabetes insipidus (CDI) is a rare clinical syndrome with an estimated frequency of 1 case in 25 000 of the population. It typically results from neoplastic, traumatic, or autoimmune destruction of the arginine vasopressin (AVP) secreting neurons [12]. A recent autopsy study that detected the viral genome and antigens in both anterior and posterior hypophysis supports the pituitary tropism of SARS-CoV-2. Moreover, compared to controls, the transcription of TSH, FSH, and LH beta-subunits genes was strongly suppressed in pituitary infected by SARS-CoV-2 [13]. Abdillah et al. showed that SARS-CoV-2 spike protein suppresses gonadotropin secretion from bovine pituitaries [14]. Gu WT et al. investigated the mRNA expression of ACE2 and angiotensin II receptor type 1 and the hormonal profiles in patients with SARS-CoV-2 infection. They conclude that SARS-CoV-2 potentially affects corticotroph cells in patients with/without pituitary disease [15]. Aside from glucocorticoid-induced adrenal insufficiency, SARS-CoV-2 can directly impair the hypothalamic–pituitary–adrenal axis by a cytopathic effect on the hypothalamus, pituitary, and adrenals, small vessels vasculitis, immune-mediated hypophysitis, microthrombotic events and glucocorticoid resistance [16]. Yavropoulou MP et al. showed that the circadian rhythm of cortisol secretion is altered even in patients with mild COVID-19, driven mainly by interleukin-6 (IL-6) hypersecretion [17]. Latent autoimmunity and polyautoimmunity are prevalent in post-COVID syndrome, while autoimmune disease may develop months after the infection [18]. Indeed, cases of CDI diagnosed six weeks and eight weeks after COVID-19 infection have been reported [7, 8]. Considering the progressive nature of some autoimmune diseases and depending on the degree of AVP secretory dysfunction, the CDI may be partial or its onset delayed.

Endocrinopathies after SARS-CoV-2 vaccination have also been described. The most common involves the thyroid. A systematic review recently reported 51 cases of SARS-CoV-2 vaccine-associated subacute thyroiditis (SAT). However, the authors concluded that SAT following SARS-Cov-2 vaccination is a very uncommon side effect. They underlined that a previous history of autoimmune thyroid disease might predispose to the occurrence of SAT after mRNA vaccines [19]. Adverse events involving the pituitary are even rarer. Roncati L et al. described a 28-year-old female who suffered from headaches for one month after the first dose of adenoviral vector-based SARS-CoV-2 vaccination. The diagnosis of pituitary apoplexy was confirmed only 69 days after the second dose [20]. In contrast, Pinar Gutierrez A et al. reported a 37-year-woman with pituitary apoplexy, diagnosed five days after a viral vector SARS-CoV-2 vaccination [21]. Besides the 2 cases of CDI following the SARS-CoV-2 vaccination mentioned above [4, 11], Morita S et al. reported a case of isolated ACTH deficiency diagnosed three weeks after the second dose of BNT162b2 SARS-CoV-2 mRNA vaccine. Three months after the onset of the event, the patient is still on glucocorticoid replacement therapy [22]. Lastly, a case of acute hypophysitis with central hypothyroidism, hypogonadism, and suppressed cortisol response to cosyntropin occurring 3 days following the second dose of mRNA-1273 SARS-CoV-2 vaccine was reported in a 51-year-old man [23].

The mechanisms that lead to endocrine disorders after SARS-CoV-2 vaccination are largely unclarified. Notably, post-COVID -19 and post-COVID-19 vaccine endocrine complications exhibit similitudes in the type of endocrine glands injured and even, but not always in the timing of occurrence. However, further studies are needed to establish whether they are different conditions. Some authors think that autoimmune/inflammatory syndrome induced by adjuvants (ASIA syndrome) may be, at least in part, responsible for the post-vaccination pituitary adverse events they observed after exposure to adjuvants in new vaccines [11, 22]. ASIA syndrome associates three major criteria, including a time frame from 8 days to 3 weeks after exposure to external stimulus (in that case, COVID-19 vaccination) for diagnosis [24]. Our patient did not meet the criteria for ASIA. Another suggested mechanism for post-SARS-CoV-2 vaccination endocrinopathies is cross-reaction between SARS-Cov-2 spike protein antibodies and the host tissue antigens [25]. The clinical presentation of our patient resembles the autoimmune hypophysitis related to immune checkpoint inhibitor therapy. Patients treated with anti-cytotoxic T-lymphocyte antigen 4 (CTLA-4) antibodies have a reported 4–20% incidence of hypophysitis. The time to onset of hypophysitis is approximately nine weeks [26].

Although the exact pathogenesis of immune checkpoint inhibitors-related hypophysitis is unknown, anti-CTLA-4 agents are known to initiate an autoimmune process that targets pituitary antigens based on pituitary autoantibodies recognizing serum thyrotrophs, corticotrophs, and gonadotrophs. Moreover, administering CTLA-4-blocking antibodies to patients who express high levels of CTLA-4 antigen in the pituitary can cause an aggressive form of hypophysitis [27]. Although we must be careful when attributing a specific complication to the SARS-CoV-2 vaccine, we may not exclude the possibility that the event was not just coincidental. Concerning our patient, several points need to be addressed. First, the differential diagnosis of hypophysitis is comprehensive, and in our initial assessment, we exclude germinoma by undetectable human gonadotropic level and pituitary neoplasm by MRI. The patient presents a typical infundibulo-neurohypophysitis with diabetes insipidus without anterior hormonal deficiencies. Second, the autoimmune etiology of lymphocytic hypophysitis may have been indicated by pituitary autoantibodies. However, their pathogenic role is unclear and not specific to hypophysitis. They are found in only 35% of patients with isolated central diabetes insipidus [10]. In contrast, anti-Rabphilin antibodies are detected in 76% of patients with infundibuloneurohypophysitis and may be helpful in the differential diagnosis of patients with central diabetes insipidus [28]. Unfortunately, these tests are not available in our hospital. Third, in the pituitary hormonal evaluation, we did not perform a cosyntropin stimulation test as a morning cortisol level > 15 µg/dL virtually excluded adrenal insufficiency [29]. Lastly, the fact that our patient has a 25-year history of Crohn's disease in prolonged remission deserves consideration. Patients with Crohn's disease have an increased risk of extra-intestinal autoimmune and inflammatory disorders compared to the general population. Granulomatous hypophysitis has been reported in patients with Crohn's but is exceptionally rare [30]. Force BK et al. reported a 43-year-old woman with a history of Crohn's disease presenting with a pituitary macroadenoma with secondary hypothyroidism and adrenal insufficiency. The patient underwent a transsphenoidal biopsy of the lesion that revealed granulomatous hypophysitis. Fifteen weeks after surgery, she developed a new onset diabetes insipidus. Under treatment with anti-tumor necrosis factor (TNF)-α polyuria resolved, and pituitary mass resolution was obtained [31]. Another case of isolated central diabetes insipidus was reported in a 39-year-old woman with Crohn's disease. The authors suggested lymphocytic hypophysitis as the cause [32]. Although we cannot exclude that the CDI in our patient could be related to her background of Crohn's disease, we believe that it is unlikely in a patient with asymptomatic disease in the context of recent SARS-CoV-2 vaccination.

In conclusion, we report a novel rare case of possible SARS-CoV-2 vaccine-related diabetes insipidus. Endocrinopathy is an infrequent adverse event of SARS-CoV-2 vaccination, although it may be underestimated. Physicians should be aware of additional and unreported clinical manifestations associated with the COVID-19 vaccine.

## Data Availability

The data that support the findings of this study are available on request from the corresponding author [AI]. The data are not publicly available because of containing information that could compromise the research participant's privacy.

## Abbreviations

COVID-19: Coronavirus disease 2019
SARS-CoV-2: Severe acute respiratory syndrome coronavirus 2
GFR: Glomerular filtration rate; TSH: Thyroid stimulating hormone
FT4: Free thyroxine; ACTH: Adrenocorticotropic hormone
IGF1: Insulin-like Growth Factor 1
ANA: Anti-nuclear antibodies
ENA: Extractable nuclear antigen
ACE2: Angiotensin-converting enzyme 2
AVPcAb: Antibodies to arginine vasopressin secreting cells
